# Etrasimod in Treatment of Ulcerative Colitis: A Comprehensive Review

**DOI:** 10.5152/tjg.2025.25148

**Published:** 2025-06-02

**Authors:** Osman Cagin Buldukoglu, Yusuf Erzin, Ayhan Hilmi Cekin, Silvio Danese

**Affiliations:** 1 Department of Gastroenterology, Antalya Training and Research Hospital, University of Health Sciences, Antalya, Türkiye; 2 Department of Gastroenterology, İstanbul University Cerrahpaşa Faculty of Medicine, İstanbul, Türkiye; 3 Gastroenterology and Endoscopy Unit, IRCCS San Raffaele Hospital, Vita-Salute San Raffaele University Faculty of Medicine and Surgery, Milan, Italy

**Keywords:** Etrasimod, treatment, ulcerative colitis

## Abstract

Ulcerative colitis (UC) is a chronic, inflammatory disease of the colon. The unpredictable, systemic, and debilitating nature of UC puts disease management and patient monitoring at a pivotal point. Despite substantial development in pharmacotherapies for UC in recent years, a significant proportion of patients either fail to respond to treatment or lose their response over the course of the disease. The backbone of disease management in UC is 5-aminosalicylic acid (5-ASA), but patients unresponsive to 5-ASA or with severe disease require advanced therapies including tumor necrosis factor-alpha inhibitors (TNFi), anti-integrins, anti-interleukins and small molecule therapy, Janus kinase (JAK) inhibitors, and S1PR modulators. This review will briefly overview the current state of medical therapeutic options in UC, with further detailing the molecular and clinical aspects of Etrasimod, a sphingosine-1-phosphate receptor (S1PR) modulator.

**Main Points**

- Ulcerative colitis is a chronic disorder with implications on both quality of life and life expectancy.
- Despite the substantial development in pharmacotherapies in recent years, a significant proportion of patients either fail to respond to treatment or lose their response over the course of the disease.
- Etrasimod, a sphingosine-1-phosphate receptor modulator, is a promising novel drug therapy for ulcerative colitis patients.

## Introduction

Ulcerative colitis (UC) is an inflammatory, chronic disease of the colon. The underlying pathogenesis is multifactorial, involving genetic factors, environmental factors, and dysregulations in the immune system, as well as alterations in the gut barrier and microbiota.^1^^,^^2^ The prevalence is estimated to be around 5 million cases worldwide, with increasing incidence.^3^ Ulcerative colitis can affect the rectum and varying parts of the colon, but extraintestinal manifestations also add to the disease burden in forms of musculoskeletal, cutaneous, hepatobiliary, and ocular involvements.^4^ Patients have a risk of experiencing acute exacerbations during the disease course, which may lead to surgical interventions and even end up in mortality.^5^^,^^6^ Apart from this relapsing-remitting and systemic nature of UC, patients are also at increased risk for colorectal cancer.^7^

The unpredictable, systemic, and debilitating nature of UC puts disease management and patient monitoring at a pivotal point. Medical treatment options form the cornerstone of UC management. Despite the substantial development in pharmacotherapies for UC in recent years, a significant proportion of patients either fail to respond to treatment or lose their response over the course of the disease.^8^ Medications are also the greatest contributors to the cost of illness in inflammatory bowel diseases (IBD), further emphasizing the requirement for new drug options for UC.^9^

This review will briefly overview the current state of medical therapeutic options in UC, with further detailing the molecular and clinical aspects of Etrasimod, a sphingosine-1-phosphate receptor (S1PR) modulator.

## Status of Current Therapeutic Options in Ulcerative Colitis

Ulcerative colitis is a chronic disorder with implications on both quality of life and life expectancy. The STRIDE (Selecting Therapeutic Targets in Inflammatory Bowel Disease) initiative has defined mucosal healing and resolution of clinical symptoms as the treatment targets in 2015.^10^ STRIDE-II statement was published in 2021, adding normalization of C-reactive protein (CRP) and fecal calprotectin, and restoration of quality of life as short-term, intermediate-term, and long-term targets, respectively, into the equation.^11^ Histological healing, disease clearance, ultrasonographic evaluation, and molecular targets were defined as treat-to-target strategies as future perspectives and potential targets.^12^

The backbone of disease management in UC is 5-aminosalicylic acid (5-ASA), but patients unresponsive to 5-ASA or with severe disease require advanced therapies including tumor necrosis factor-alpha inhibitors (TNFi), anti-integrins, anti-interleukins and small molecule therapy, Janus kinase (JAK) inhibitors, and S1PR modulators.^13^ Corticosteroids and conventional immunomodulators (e.g. azathioprine, methotrexate) can also be utilized during the disease course.

5-Aminosalicylic acid is still the preferred drug, both for induction and maintenance therapy, in patients with mild-to-moderate UC.^14^ 5-ASA is generally accepted to be safer than immunomodulators and biologics. Adherence to therapy is an important component of 5-ASA therapy, and efforts to address this issue include the development of new formulations and the implementation of patient education.

Conventional immunomodulators, while having lost some affection in the era of biologics and small molecule therapy, are still valuable options in certain scenarios. Considering the efficacy and unfavorable long-term exposure safety profile, traditional immunomodulators can be used in patients with mild disease who are unresponsive to 5-ASA or fail to maintain a corticosteroid-free remission state.^15^

Corticosteroids are strong options in severe UC. They are also effective in remission induction of patients with mild or moderate disease who are unresponsive to 5-ASA.^16^ Long-term corticosteroid use comes with serious negative impacts for both the patient and the healthcare system, hence poorly absorbed forms such as budesonide are gaining favor, especially in patients with mild-to-moderate UC.^17^

The introduction of TNFi as a therapeutic option for IBD in the 1990s was a breakthrough for both the patients and the healthcare team. Effective against both colonic disease involvement and extraintestinal manifestations, TNFi comprises an important part of IBD management.^18^^,^^19^ Anti-α4β7 integrin Vedolizumab is a gut-selective treatment option.^20^^,^^21^ Anti-IL12/23 drugs, with Ustekinumab being the first and most commonly used one, are shown to be effective and safe in UC patients.^22^ Tofacitinib, filgotinib, and upadacitinib are JAK inhibitors which are taken orally and effective in the treatment of moderate-to-severe UC.^23^ S1PR modulators block lymphocyte egress from secondary lymphoid organs, Etrasimod, a member of this drug family, being the main topic of this review.^24^

## Etrasimod

Etrasimod is a selective S1PR modulator, targeting S1PR1, S1PR4, and S1PR5, which is taken by oral route. Approved in the United States in October 2023 and by the European Medicines Agency in December 2023 for the treatment of patients with moderate-to-severe UC, the low rates of immunogenicity and lower cost compared to biologics put the molecule in the spotlight as a therapeutic option.^25^^,^^26^

### Sphingosine-1-Phosphate: Molecular Structure and Mechanism of Action

Sphingosine-1-phosphate is a membrane-derived, bioactive lysophospholipid that exerts its functions through 5 G protein-coupled S1P receptors.^27^ This active sphingolipid metabolite is shown to partake as a regulator in many critical physiological and pathophysiological pathways in the cardiovascular, nervous, and immune systems, including carcinogenesis.^28^ Since its first discovery as a signaling molecule back in 1993, numerous studies have led to the identification of various processes related to S1P and S1PR, eventually revealing the role of S1P in the migration of immune cells.^29^

**Figure 1.**
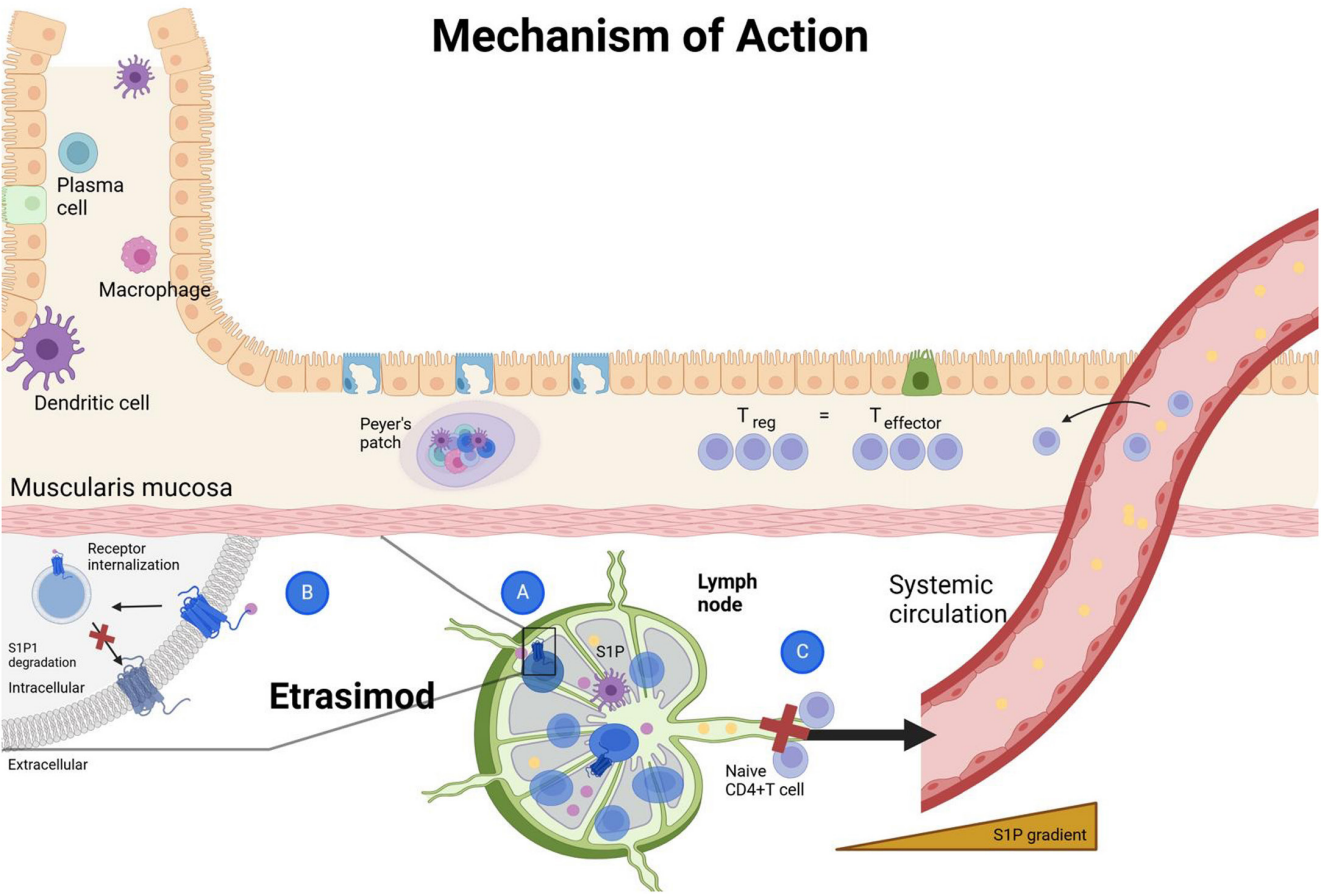
Etrasimod – mechanism of action. A: Etrasimod – S1PR interaction. B: Internalization of S1PR. C: Blockage of lymphocyte egress. S1PR: Sphingosine-1-phosphate receptor.

**Figure 2.**
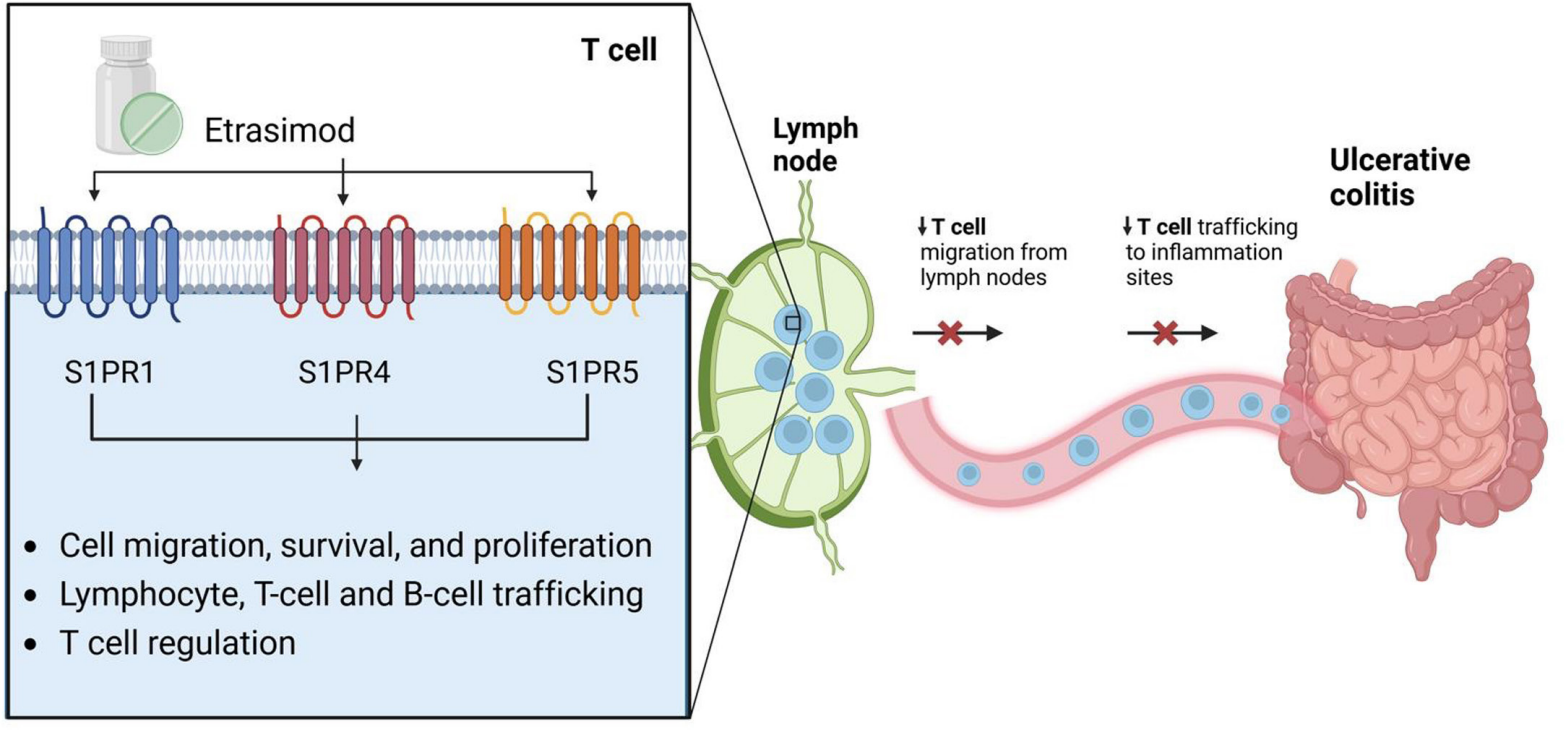
Selective S1PR modulation leading to reduced T cell trafficking. S1PR: Sphingosine-1-phosphate receptor.

Phosporylation of sphingosine, by sphingosine kinases 1 and 2, leads to the formation of S1P. Five distinct receptors for S1P are currently identified, namely S1PR1 to S1PR5. These receptors are shown to be expressed on innate immune cell types. S1PR1 is expressed in every cell partaking in innate immunity including macrophages, neutrophils, dendritic cells, monocytes, eosinophils, mast cells, and natural killer cells. S1PR4 and S1PR5, which are the selective targets for Etrasimod along with S1PR1, show varying expression on immune cells. S1PR4 is expressed on every innate immune cell subtype except natural killer cells, while S1PR5 is expressed on natural killer cells and patrolling monocytes.^30^ This wide array of expression on immune cell subtypes translates into S1P and its receptors being a prominent actor regulating numerous pathways including migration of immune cells, cellular and vascular integrity, angiogenesis, and inflammation. The dysregulated immune response is the backbone of IBD pathogenesis, hence putting S1P and its receptors in the spotlight as a therapeutic target (Figures 1 and 2).^31^

Etrasimod, a once-daily, oral selective S1PR1-4-5 modulator, is a novel molecule approved for use in patients with moderate-to-severe UC.

### Efficacy of Etrasimod in Treatment of Ulcerative Colitis

Etrasimod is a small molecule drug with a low molecular weight that partially and reversibly inhibits lymphocyte egress from secondary lymphoid organs. As an orally administered small molecule, it offers the advantages of high bioavailability, rapid absorption, lack of immunogenicity, and a short half-life.^32^ This makes it a preferable therapeutic option for patients who are deterring from self-injections or intravenous treatments, as well as in patients who may need a rapid discontinuation.

The efficacy and safety of Etrasimod were investigated in a number of studies. A phase 2, randomized, multi-center trial in moderately to severely active UC patients included 156 patients who were split into 3 groups and given a placebo, once-daily etrasimod 1 mg or once-daily etrasimod 2 mg. The study population comprised adult patients with modified Mayo Clinic scores (MCS) of 4-9, endoscopic subscores of 2 or more, and rectal bleeding subscores of 1 or more. Prednisone equal to or less than 10 mg/d and budesonide equal to or less than 9 mg/d were permitted at a stable dose, whereas agents administered via rectal route and biologics were discontinued 2 weeks and 60 days before the study, respectively. The primary endpoint was improvement from baseline in modified MCS at week 12. Secondary endpoints were designated as the proportion of patients with endoscopic improvement at week 12, improvement in endoscopy findings and rectal bleeding according to MCS, and improvement in the total MCS. At week 12, patients in the etrasimod 2 mg group met all the primary and secondary endpoints. The endoscopic improvement rate was 41.8% in patients on etrasimod 2 mg compared to 17.8% receiving placebo.^33^

Two independent, randomized, double-blind, and placebo-controlled phase 3 studies further evaluated the role of etrasimod as induction and maintenance therapy for UC. These 2 trials, ELEVATE UC 12 and ELEVATE UC 52, included adult patients with moderate to severely active UC who also had an inadequate response, loss of response, or intolerance to at least 1 therapy approved for UC. ELEVATE UC 12 and ELEVATE UC 52 studies comprised 354 and 433 patients, respectively. About 238 out of 354 patients were assigned to etrasimod in ELEVATE UC 12, whereas 289 out of 433 patients were assigned to etrasimod in ELEVATE UC 52. ELEVATE UC 12 assessed induction at week 12, while ELEVATE UC 52 comprised a 40-week maintenance period following a 12-week induction period. Etrasimod was found to be effective and well tolerated in both induction and maintenance therapy for patients with moderately to severely active UC. 25% of patients (n = 55) in the etrasimod group had clinical remission compared to 15% of patients (n = 17) in the placebo group at week 12 in ELEVATE UC 12 (*P* = .026). 32% of patients (n = 88) in the etrasimod group had clinical remission compared to 7% of patients (n = 9) in the placebo group at week 52 in ELEVATE UC 52 (*P* < .0001).^34^

The effect of age on the efficacy and safety of etrasimod in patients with ulcerative colitis was investigated with a post hoc analysis of data from the ELEVATE UC clinical program. 787 patients were enrolled in this study. About 420 patients (53.4%) were below the age of 40, whereas 276 patients (35.1%) were ages between 40 and 59, and 91 patients (11.6%) were equal to or above the age of 60. Significant clinical benefit was observed in the etrasimod group compared to the placebo group regardless of age. Etrasimod 2 mg had a consistent safety profile across all age groups, with no difference in adverse event rates.^35^ Another recently published study comprised 743 patients with moderately active (n = 525, 70.7%) and severely active (n = 218, 29.3%) UC, investigating the efficacy of etrasimod in UC. Etrasimod showed higher rates of clinical response and reduction in disease activity compared to placebo at week 12.^36^

Patients with isolated proctitis (with less than 10 cm of rectal involvement) were evaluated with a post hoc analysis from ELEVATE UC 12 and ELEVATE UC 52 trials for the efficacy and safety of etrasimod 2 mg. Clinical remission rates were 42.9% and 13.6% at week 12 and 44.4% and 11.1% at week 52 for etrasimod 2 mg and placebo respectively.^37^ Another post hoc analysis was performed to evaluate the efficacy of etrasimod as monotherapy versus concomitant use of 5-ASA and/or corticosteroids. In this analysis, no additional benefit was observed in patients receiving concomitant 5-ASA or corticosteroids compared to patients receiving etrasimod monotherapy.^38^ An important study evaluated the efficacy of etrasimod in patients with prior biologic/JAK inhibitor exposure, utilizing the data gathered in ELEVATE UC 12 and ELEVATE UC 52 trials. Etrasimod was found to be superior to placebo in both biologic-/JAK inhibitor naive and experienced patients, with treatment effect being more consistent in the treatment-naive group. Prior anti-integrin exposure was found to have a negative impact on clinical remission rates with etrasimod.^39^ Parallel to these findings, the American Gastroenterological Association guideline published in December 2024 put etrasimod in the “higher efficacy medications” group for first-line therapy in advanced therapy-naive patients.^40^ A recent network meta-analysis which included 24 randomized controlled trials comprising 15 therapies and 8874 patients ranked etrasimod 2 mg per day as the highest in achieving remission and histologic improvement following induction in patients with moderate-to-severe UC.^41^

Biomarkers are vital in monitoring disease course and treatment response in UC. Fecal calprotectin (fCAL) and high-sensitivitiy C-reactive protein (hsCRP) levels of patients included in ELEVATE UC program were evaluated. Both fCAL and hsCRP levels showed a decline with etrasimod treatment and receiver operating characteristics curves revealed a prognostic correlation between fCAL during the induction period and short and long-term treatment response.^42^ The findings of this study was in line with a post hoc analysis of the phase 2 OASIS trial, which also revealed fCAL and CRP as noninvasive biomarkers, as well as revealing the efficacy of etrasimod in achieving endoscopic improvement-histologic remission.^43^

### Safety Profile

The safety of novel drug therapies is an important factor in the decision process of healthcare team. As with any medication, there are important points that should be kept in mind for etrasimod therapy.

Adverse events related to and long-term safety of etrasimod treatment have been investigated in an open-label extension (OLE) of the OASIS study, in which patients received etrasimod 2 mg per day for an additional 34 to 40 weeks. Overall, 112 patients were included in the OLE, with 42 being in the placebo group, 38 in the etrasimod 1 mg group, and 32 in the etrasimod 2 mg group previously. Ten of 112 patients (9%) in the etrasimod 2 mg group discontinued the study drug, with 8 having worsening of UC, and 1 each with headache and atrial fibrillation. Three patients in the etrasimod 2 mg group experienced first-degree atrioventricular (AV) block. Two of these patients were previously in the placebo group and the other 1 received etrasimod 2 mg in the OASIS study. None of the patients discontinued therapy due to AV block, with 2 being considered clinically insignificant and 1 being considered mild. There were no serious infections related to treatment. Two cases of herpes zoster were observed but neither of the patients required cessation of treatment.^44^

Cardiovascular events in patients receiving etrasimod therapy were investigated in a recently published paper.^45^ Cardiovascular treatment-emergent adverse events (TAEAs) were infrequent (2.6% per adverse event). Incidence rates (IRs) per 100 patient-years were 3.85 for bradycardia and 1.40 for AV block in patients receiving etrasimod compared to 0 for the placebo group for both adverse events. The IR for hypertension was 5.31 in the etrasimod group and 3.40 in the placebo group. All bradycardia and hypertension events were non-serious. One serious AV block type 1, second-degree, was observed in the etrasimod group; as well as 1 case of each for coronary artery disease and cerebrovascular disease.

Infections, which are a major safety concern in UC patients, were investigated in events from ELEVATE UC program. The analysis revealed similar IR for patients receiving etrasimod 2 mg per day (18.8%) versus placebo (17.7%). Serious infections occurred in 3 patients (0.6%) on etrasimod therapy and 5 patients (1.9%) on placebo. Two herpes zoster events were observed in both groups which were all localised and non-serious cases, and 1 opportunistic infection was recorded in each group. There were no serious, severe, or opportunistic infections in patients with an absolute lymphocyte count below 200/mm^3^, and no deaths occurred in the study population.^46^ Advanced age was also not found to be associated with additional risks for etrasimod treatment.^35^

Etrasimod is contraindicated in patients who have experienced myocardial infarction, unstable angina pectoris, stroke, transient ischemic attack, decompensated heart failure requiring hospitalization, or Class III or IV heart failure. It is also contraindicated in patients with a history or presence of Mobitz type II second-degree or third-degree AV block, sick sinus syndrome, or sino-atrial block unless the patient has a functioning pacemaker.

## Conclusion

Etrasimod is an easy-to-use small molecule with high efficacy in patients with moderate-to-severe UC, especially in advanced treatment-naive patients as a first-line therapy. Selective modulation of S1P receptors also creates a favorable safety profile for this novel molecule. Real world evidence will add up on the current knowledge and experience on this therapeutic option, hopefully strengthening the armamentarium against UC, which is a burdensome disorder for both the patient and healthcare team.

## Data Availability

The data that support the findings of this study are available on request from the corresponding author.
